# HTA and Reimbursement Status of Metastatic Hormone‑Sensitive Prostate Cancer, Non-Metastatic Castration-Resistant Prostate Cancer, and Metastatic Castration-Resistant Prostate Cancer Treatments in Europe: A Patient Access Landscape Review

**DOI:** 10.36469/001c.75208

**Published:** 2023-06-20

**Authors:** Goran Bencina, Elina Petrova, Demet Sönmez, Sonia Matos Pereira, Ioannis Dimitriadis, Stina Salomonsson

**Affiliations:** 1 Center for Observational and Real-World Evidence, MSD, Madrid, Spain; 2 Market Access and Pricing, MSD, Sofia, Bulgaria; 3 Center for Observational and Real-World Evidence, MSD, Stockholm, Sweden; 4 Market Access, MSD, Lisbon, Portugal; 5 Medical Affairs, MSD, Athens, Greece

**Keywords:** prostate cancer, anticancer drugs, patient access, reimbursement decision, ESMO-MCBS, HTA

## Abstract

**Background:** Prostate cancer is the second most common cancer in men, with up to one-third of men being diagnosed in their lifetime. Recently, novel therapies have received regulatory approval with significant improvement in overall survival for metastatic castration-resistant prostate cancer, metastatic hormone-sensitive prostate cancer, and nonmetastatic castration-resistant prostate cancer. To improve decision-making regarding the value of anticancer therapies and support standardized assessment for use by health technology assessment (HTA) agencies, the European Society for Medical Oncology (ESMO) has developed a Magnitude of Clinical Benefit Scale (MCBS).

**Objective:** This review aimed to map HTA status, reimbursement restrictions, and patient access for 3 advanced prostate cancer indications across 23 European countries during 2011-2021.

**Methods:** HTA, country reimbursement lists, and ESMO-MCBS scorecards were reviewed for evidence and data across 26 European countries.

**Results:** The analysis demonstrated that only in Greece, Germany, and Sweden was there full access across all included prostate cancer treatments. Treatments available for metastatic castration-resistant prostate cancer were widely reimbursed, with both abiraterone and enzalutamide accessible in all countries. In 3 countries (Hungary, the Netherlands, and Switzerland), there was a statistically significant difference (*P*<.05) between status of reimbursement and ESMO-MCBS “substantial benefit” (score of 4 or 5) vs “no substantial benefit” (score <4).

**Conclusion:** Overall, the impact of the ESMO-MCBS on reimbursement decisions in Europe is unclear, with significant variation across the countries included in this review.

## INTRODUCTION

Prostate cancer is the second most common cancer in men, with up to one-third of men being diagnosed in their lifetime.^1^ In 2020, it was estimated that 1.4 million new cases and 375 000 deaths would occur worldwide, making it one of the leading causes of death in men globally.^2^ Compared with other cancers, prostate cancer typically progresses slowly and, if diagnosed early, may be managed successfully with radiation, surgery, or hormone therapy.

There are multiple subclassifications of prostate cancer. Metastatic cancer, commonly referred to as advanced cancer, is categorized as cancer spreading beyond the point of origin. Cancer can enter the bloodstream or lymphatic system, travel to different regions of the body, and grow in nearby areas. Up to one-third of patients will progress to a metastatic form of prostate cancer, with the poorest prognosis resulting from a diagnosis with metastatic castration-resistant prostate cancer (mCRPC).^3^ Preceding mCRPC is metastatic hormone-sensitive prostate cancer (mHSPC), where the cancer has spread beyond the prostate but can be treated with hormone therapy.^4^ Nonmetastatic castration-resistant prostate cancer (nmCRPC) is diagnosed as a rising prostate-specific antigen concentration, despite castrate levels of testosterone following treatment with hormone therapy or orchiectomy, the removal of one or both testicles.^5^ In nmCRPC, the cancer has not spread beyond the prostate.^5^

In recent years, novel innovative therapies and treatment options have become available for patients with prostate cancer. Treatment options for mCRPC have progressed beyond chemotherapy with docetaxel to novel hormonal agents (NHA), such as abiraterone and enzalutamide, approved by the European Medicines Agency (EMA) in 2011 and 2013, respectively (**Table 1**).^6^ At the time of approval, these NHAs were originally recommended as second-line (2L) treatments. This recommendation has evolved to introduce these novel therapies earlier in treatment in case of failure to conventional androgen deprivation therapy (ADT).^6^ Polyadenosine diphosphate–ribose polymerase inhibitors have also been used in patients with breast cancer gene (BRCA) 1/2-mutation–positive mCRPC.^7^

**Table 1.** European Medical Agency–approved Therapies for Prostate Cancer

**Drug Name**	**EMA Approval Date**	**Indication**
mHSPC		
ADT + abiraterone	15 Nov 2017	Abiraterone is indicated with prednisone or prednisolone for the treatment of newly diagnosed high-risk mHSPC in adult men in combination with ADT.^8^
ADT + enzalutamide	30 April 2021	Enzalutamide is indicated for the treatment of adult men with mHSPC in combination with ADT.^9^
ADT + apalutamide	27 Jan 2020	Apalutamide is indicated in adult men for the treatment of mHSPC in combination with ADT.^10^
mCRPC (1st-line)		
Abiraterone	18 Dec 2012	Abiraterone is indicated with prednisone or prednisolone for: the treatment of mCRPC in adult men who are asymptomatic or mildly symptomatic after failure of ADT in whom chemotherapy is not yet clinically indicated.^8^
Enzalutamide	28 Nov 2014	Enzalutamide is indicated for the treatment of adult men with mCRPC who are asymptomatic or mildly symptomatic after failure of ADT in whom chemotherapy is not yet clinically indicated.^9^
mCRPC (2nd-line/postchemotherapy)		
Abiraterone	5 Sept 2011	Abiraterone is indicated with prednisone or prednisolone for the treatment of mCRPC in adult men whose disease has progressed on or after a docetaxel-based chemotherapy regimen.^8^
Cabazitaxel	17 March 2011	Cabazitaxel in combination with prednisone or prednisolone is indicated for the treatment of adult patients with mCRPC previously treated with a docetaxel-containing regimen.^11^
Enzalutamide	21 June 2013	Enzalutamide is indicated for the treatment of adult men with mCRPC whose disease has progressed on or after docetaxel therapy.^9^
Olaparib	3 Nov 2020	Olaparib is indicated as monotherapy for the treatment of adult patients with mCRPC and BRCA 1/2 mutations (germline and/or somatic) who have progressed following prior therapy that included an NHA.^12^
nmCRPC		
ADT + apalutamide	14 Jan 2019	Apalutamide is indicated in adult men for the treatment of nmCRPC who are at high risk of developing metastatic disease in combination with ADT.^10^
ADT +darolutamide	27 March 2020	Darolutamide is indicated for the treatment of adult men with nmCRPC who are at high risk of developing metastatic disease in combination with ADT.^13^
ADT + enzalutamide	23 Oct 2018	Enzalutamide is indicated for the treatment of adult men with high-risk nmCRPC in combination with ADT.^9^
mCRPC with symptomatic bone metastases		
Radium Ra 223 dichloride	14 Nov 2013	Radium Ra 223 dichloride monotherapy or in combination with LHRH analog is indicated for the treatment of adult patients with mCRPC, symptomatic bone metastases and no known visceral metastases, in progression after ≥2 prior lines of systemic therapy for mCRPC (other than LHRH analogs), or ineligible for any available systemic mCRPC treatment.^14^

Abbreviations: ADT, androgen deprivation therapy; BRCA, breast cancer gene; EMA, European Medical Agency; LHRH, luteinizing hormone–releasing hormone; mCRPC, metastatic castration-resistant prostate cancer; mHSPC, metastatic hormone-sensitive prostate cancer; NHA, novel hormonal agent; nmCRPC, nonmetastatic castration-resistant prostate cancer.

Following 7 decades of treatment exclusively with ADT in patients with mHSPC, the drug landscape has begun to evolve in the past 5 years.^3^ Clinical trials examining the impact of docetaxel chemotherapy found significant improvement in overall survival compared with ADT alone.^3^ The initiation of innovative therapies sparked the introduction of NHAs (abiraterone, enzalutamide, and apalutamide) in this disease stage.^3^

Patients with nmCRPC are at risk of progressing to metastatic cancer. In efforts to mitigate risk, enzalutamide, apalutamide, or darolutamide are prescribed in addition to ADT.^5^

Despite the numerous therapies currently available for patients with prostate cancer, equity in the availability of treatments varies across European countries. Following approval for use by the EMA, individual countries assess each therapy for acceptability into national health systems. A key consideration for recommendation by healthcare policymakers and decision-makers are the associated costs of treatments. Negotiations between pharmaceutical companies and decision-makers are typically not timely and can delay patient access to life-saving treatment. In 2015, the European Society for Medical Oncology (ESMO) launched the ESMO–Magnitude of Clinical Benefit Scale (ESMO-MCBS) to improve decision-making regarding the value of anticancer therapies and to promote the access to high-value cancer treatments.^15^ The purpose of the scale was to promote scientific integrity by reducing bias in interpretation of data and analyses and to reduce “hype” surrounding novel therapies. In turn, it was anticipated to provide robust validation with strict adherence to standards for “accountability for reasonableness” and to provide a reliable and fair evaluation of benefit to assist in resource allocation decisions.^15^ The scorecard classifies drugs with grade A, B, or C in a curative setting or 1 through 5 in a noncurative setting. Treatments with a grade of A-B or 4-5 are considered to have “substantial benefit.”^15^

Previous studies have investigated and quantified the economic burden associated with prostate cancer and associated cost of treatment.^16–18^ This study aimed (1) to provide an overview of health technology assessment (HTA) decisions, reimbursement status, restrictions, and patient access of mHSPC, nmCRPC, and mCRPC treatments across selected European countries and (2) to understand the impact of ESMO-MCBS on the reimbursement decisions and thereby the impact on patient access to advanced prostate cancer treatments. Due to potential variation in treatment cost and pricing systems across the selected countries, this paper reports on reimbursement decisions as opposed to cost of treatment.

## METHODS

### Search Strategy

HTA agency websites with country reimbursement status of the drug lists and ESMO-MCBS scorecards websites^18–45^ were searched from June 7, 2022, for evidence and data to use in this study. The agency website list was compiled based on information from several sources including the publicly available lists of HTA agencies from the International Network of Agencies for Health Technology Assessment and European Network for Health Technology Assessment. Searches were conducted based on molecule names included in the analysis (**Table 2**). Data were obtained and analyzed between June 7 and October 31, 2022, for records of molecules that received regulatory approval from 2011 to the time of search. Countries included in this review were Austria, Belgium, Bulgaria, Croatia, Czech Republic, Denmark, Estonia, Finland, France, Germany, Greece, Hungary, Italy, Latvia, Lithuania, the Netherlands, Norway, Poland, Portugal, Romania, Slovakia, Slovenia, Spain, Sweden, Switzerland, and the United Kingdom (UK).

**Table 2.** Eligibility Criteria for Inclusion

Population(s)	Adult patients with mHSPC, nmCRPC, and mCRPC
Interventions	NHA including: Abiraterone Enzalutamide Apalutamide Darolutamide Chemotherapy: Cabazitaxel PARPi: Olaparib Ra 223 dichloride
Comparisons	Not applicable
Outcomes	HTA decisions and reimbursement status Reimbursement restrictions ESMO-MCBS scorecards for drugs in the scope
Time	Molecules with regulatory approval from 2011 to 2021
Study design	All types of appraisal reports, including extensions of recommendations, report updates, and new reports ESMO-MCBS scorecards

Abbreviations: ESMO-MCBS: European Society for Medical Oncology-Magnitude of Clinical Benefit Scale; HTA: health technology assessment; mCRPC: metastatic *castration*-*resistant* prostate cancer; mHSPC: metastatic hormone-sensitive prostate cancer; NHA, new hormonal agents; nmCRPC: nonmetastatic castration-resistant prostate cancer; PARPi: polyadenosine diphosphate–ribose polymerase inhibitor.

### Eligibility Criteria and Screening Methods

Identified materials and evidence were reviewed for inclusion based on prespecified eligibility criteria detailed in **Table 2**. All type of appraisal reports, including extensions of recommendations, updates of reports, and new reports were included. Evidence was limited to patients with mHSPC, nmCRPC, and mCRPC, exclusively. Treatments included those currently approved by the EMA (**Table 1**) for targeted indications. Treatment with docetaxel was excluded from the analysis as it is a dated cytostatic used widely in oncology treatment. In many of the countries, there were no HTA/reimbursement processes for the use of docetaxel in prostate cancer. Documents pertaining to molecules with regulatory approval granted prior to 2011 and relating to countries outside Europe were excluded.

Two reviewers independently reviewed the reimbursement status, restrictions, and ESMO-MCBS scorecards and extracted data into a prespecified data extraction grid in Microsoft Excel® (**Supplementary Appendix**) against the eligibility criteria in duplicate. Any disagreements were referred to a third reviewer.

### Data Extraction and Statistical Analyses

Data reporting on HTA status, reimbursement decisions, and ESMO-MCBS scorecards were obtained and analyzed from June 7 to October 31, 2022. Data were extracted and summarized into a predefined data extraction grid. A 2-sided Fisher’s exact test was used to compare the ESMO-MCBS scores between the drugs approved for reimbursement and nonapproved drugs using GraphPad Prism version 9.4.1.

## RESULTS

### Identification of Evidence

Data for ESMO-MCBS score, HTA and reimbursement status, and restrictions of patient access of mHSPC, nmCRPC, and mCRPC treatments were reported in 23 of the 26 selected European countries (Austria, Belgium, Bulgaria, Croatia, Czech Republic, Denmark, Finland, France, Germany, Greece, Hungary, Italy, the Netherlands, Norway, Poland, Portugal, Romania, Slovakia, Slovenia, Spain, Sweden, Switzerland, and the UK).

### Reimbursement and HTA Status

When comparing countries reimbursement status, this review found that all treatments for mHSPC, nmCRPC, and mCRPC included in the analysis are reimbursed only in Germany, Sweden, and Greece (**Figure 1**). Significant disparity exists in the availability of treatments across countries. For example, Denmark, Finland, Poland, and Slovakia do not provide unrestricted reimbursement for any treatment, while Bulgaria, France, Germany, the Netherlands, and Norway provide unrestricted reimbursement of 10 or more treatments.

**Figure 1.** Reimbursement and HTA Status of mHSPC, nmCRPC, and mCRPC Treatments in Selected European Countries

**Treatment**	**ESMO-⁠MCBS**	**Austria**	**Belgium**	**Bulgaria**	**Croatia**	**Czech Republic**	**Denmark**	**Finland**	**France**	**Germany**	**Greece**	**Hungary**	**Italy**	**The Netherlands**	**Norway**	**Poland**	**Portugal**	**Romania**	**Slovakia**	**Slovenia**	**Spain**	**Sweden**	**Switzerland**	**UK**
mHSPC																								
ADT + abiraterone	4	R	R	Y	R	R	N	R	Y	Y	Y	Y	Y	Y	N	N	N	Y	R	N	R	Y	R	N
ADT + enzalutamide	4	R	N	Y	R	N	N	R	N	Y	Y	N	N	Y	N	N	N	N	N	Y	N	Y	R	Y
ADT + apalutamide	4	R	R	Y	R	R	N	R	Y	R	Y	N	N	Y	Y	N	Y	Y	R	Y	R	R	Y	N
mCRPC (1st-line)																								
Abiraterone	4	R	R	Y	Y	R	R	R	Y	Y	Y	Y	R	Y	Y	R	Y	Y	R	Y	R	Y	R	Y
Enzalutamide	4	R	R	Y	R	R	R	R	Y	Y	Y	Y	R	Y	Y	R	R	Y	R	Y	R	R	R	Y
mCRPC (2nd-line/postchemotherapy)																								
Abiraterone	4	R	R	Y	R	R	R	R	Y	Y	Y	R	R	Y	Y	R	Y	N	N	Y	R	Y	R	R
Cabazitaxel	3	Y*	R	Y	R	R	R	Ya	Y	Y	Y	N	R	Y	Y	N	Y	Y	N	Y	R	R	R	R
Enzalutamide	4	R	R	Y	R	R	R	R	Y	Y	Y	R	R	Y	Y	R	Y	Y	N	Y	R	Y	R	Y
Olaparib	3	A	R	N	N	N	R	N	Y	Y	Y	N	N	Y	N	N	N	Y	N	N	N	R	N	N
nmCRPC																								
ADT + apalutamide	3	R	R	Y	R	R	R	R	Y	Y	Y	N	Y	N	Y	R	R	Y	R	Y	R	R	N	N
ADT + darolutamide	3	R	R	Y	R	R	R	R	Y	Y	Y	N	Y	N	Y	R	R	N	N	Y	R	Y	N	Y
ADT + enzalutamide	3	Y	R	Y	R	N	R	R	Y	Y	Y	N	N	N	Y	R	N	N	N	Y	N	Y	N	N
mCRPC with symptomatic bone metastases																								
Radium Ra 223 dichloride	5	Y	R	N	R	R	R	Ya	Y	Y	Y	Y	R	Y	Y	R	Y	N	N	R	Y	R	R	R

Abbreviations: A, available but not reimbursed; ADT, androgen deprivation therapy; ESMO-MCBS, European Society for Medical Oncology–Magnitude of Clinical Benefit Scale; mCRPC, metastatic castration-resistant prostate cancer; mHSPC, metastatic hormone-sensitive prostate cancer; N, not reimbursed; nmCRPC, nonmetastatic castration-resistant prostate cancer; R, reimbursed with restrictions; Y, reimbursed.

^a^Countries have not received national reimbursement for these treatments, but it is funded at a hospital level.

In mHSPC, patients in Denmark and Poland do not have access to any of the available molecules. Treatment with ADT + enzalutamide was reimbursed less often than any other treatment for mHSPC (n=12). Inconsistent recommendations are provided despite an ESMO-MCBS score of 4, “substantial benefit” for all treatments. In general, Finnish HTA bodies do not grant reimbursement for combination products but will reimburse individual products if they meet requirements for outpatient care products. At the time of this review, ADT + enzalutamide was under evaluation in Norway; thus, changes in reimbursement status may occur in coming months.

First-line treatments available for mCRPC are more widely reimbursed than those for mHSPC, with both abiraterone and enzalutamide fully reimbursed or reimbursed with restrictions in all countries. Both treatments have received an ESMO-MCBS score of 4, indicating substantial benefit. In 2L therapies for mCRPC, olaparib is not reimbursed in 14 of 23 countries. Although olaparib is not reimbursed in Austria, it is considered on a case-by-case basis by the treating physician. Cabazitaxel is funded by hospital budgets in Finland and Austria.

None of the 3 available nmCRPC treatments are reimbursed in Finland, Hungary, and the Netherlands. Over half (n=13) of the countries provide full reimbursement or with restrictions across concomitant administration of ADT and apalutamide, darolutamide, or enzalutamide.

For patients with mCRPC with symptomatic bone metastases, radium Ra 223 dichloride is fully reimbursed in 9 countries, reimbursed with restrictions in 10 countries, and not reimbursed in Bulgaria, Romania, or Slovakia. In Finland, radium Ra 223 dichloride is funded by hospital budgets. Compared with all treatments identified in this review, radium Ra 223 dichloride is ranked the highest on the ESMO-MCBS, with a score of 5.

Five treatment options are scored 3 on the ESMO-MCBS (cabazitaxel, olaparib, ADT + apalutamide, ADT + darolutamide, and ADT + enzalutamide). Despite receiving the same score and perceived clinical benefit, significant variation exists in reimbursement status. Olaparib received no reimbursement in 14 out of 23 countries, with a further 3 restricting reimbursements. In contrast, ADT + apalutamide did not receive reimbursement in 4 countries, with another 11 countries adding restrictions to reimbursement.

### Reimbursement Restrictions

The findings of this review indicated that in 6 countries (Bulgaria, France, Greece, the Netherlands, Norway, and Romania), no further reimbursement restrictions were made on treatments for mHSPC, nmCRPC, or mCRPC vs the EMA label. All other countries imposed a form of reimbursement restriction compared with the label, and Denmark included additional reimbursement restrictions for all reimbursed products (**Table 3**). In some countries, such as Spain and Italy, it was also noted that there may be variations in the restrictions applied within the country, depending on the region being assessed.

**Table 3.** Comparison Between Number of Reimbursements and Number of Restrictions by Country

**Treatment**	**No. of Countries With Reimbursement**	**Countries With Restrictions**	**No. of Countries With Restrictions**
mHSPC			
ADT + abiraterone	17	Austria, Belgium, Croatia, Czech Republic, Finland, Slovakia, Spain, Switzerland	8
ADT + enzalutamide	11	Austria, Croatia, Finland, Switzerland	4
ADT + apalutamide	18	Austria, Belgium, Croatia, Czech Republic, Finland, Germany, Slovakia, Spain, Sweden, Switzerland	10
mCRPC (1L)			
Abiraterone	23	Austria, Belgium, Croatia, Denmark, Finland, Italy, Poland, Slovakia, Spain, Switzerland	10
Enzalutamide	23	Austria, Belgium, Croatia, Czech Republic, Denmark, Finland, Italy, Poland, Portugal, Slovakia, Spain, Sweden, Switzerland	13
mCRPC (2L/postchemotherapy)			
Abiraterone	21	Austria, Belgium, Croatia, Czech Republic, Denmark, Finland, Hungary, Italy, Poland, Spain, Switzerland, UK	12
Cabazitaxel	20	Belgium, Croatia, Czech Republic, Denmark, Italy, Spain, Sweden, Switzerland, UK	9
Enzalutamide	22	Austria, Belgium, Croatia, Czech Republic, Denmark, Hungary, Italy, Poland, Spain, Switzerland	10
Olaparib	8	Belgium, Denmark, Switzerland	3
nmCRPC			
ADT + apalutamide	19	Austria, Belgium, Croatia, Czech Republic, Denmark, Finland, Poland, Portugal, Slovakia, Spain, Sweden	11
ADT + darolutamide	18	Austria, Belgium, Croatia, Czech Republic, Denmark, Finland, Poland, Portugal, Spain	9
ADT + enzalutamide	13	Belgium, Croatia, Denmark, Finland, Poland	5
mCRPC with symptomatic bone metastases			
Radium Ra 223 dichloride	20	Belgium, Croatia, Czech Republic, Italy, Poland, Slovenia, Sweden, Switzerland, UK	9

Abbreviations: 1L, first-line; 2L, second-line; ADT, androgen deprivation therapy; mCRPC, metastatic castration-resistant prostate cancer; mHSPC, metastatic hormone-sensitive prostate cancer; nmCRPC, nonmetastatic castration-resistant prostate cancer.

These restrictions were further evaluated and categorized as (1) country-specific for all types and lines of prostate cancer and (2) common across the countries. Full details of country-specific restrictions are included in the **Supplementary Online Materia**l.

A number of country-specific restrictions applied across all types and lines of prostate cancer. This included those in Austria, where all the restrictions are defined as “yellow box (reimbursed with prior validation of chief-doctor)”; Finland, where all the restrictions are “reimbursement requested and granted based on clinician’s written statement”; and Denmark, where all the restrictions stated “mandatory to use drug with lowest cost.”

Among mHSPC, nmCRPC, and mCRPC treatments, the most common restriction is to patients with an ECOG score of 0-1. In mHSPC, the restriction to ECOG 0-1 performance status is included in 3 countries (Germany, Czech Republic, and Croatia). In first-line mCRPC, this applies in 4 countries (Portugal, Italy, Spain, and Czech Republic), for 2L mCRPC in 3 countries (UK, Poland, and Spain) and in nmCRPC the restriction for ECOG 0-1 performance status is included in 5 countries (Portugal, Poland, Spain, Czech Republic, and Croatia) (see **Supplementary Appendix**).

### ESMO-MCBS Scorecards

When comparing the mean ESMO-MCBS scores of treatments considered to provide “substantial benefit” (score of 4-5) vs a score of less than 4 (“no substantial benefit”) across reimbursed and nonreimbursed treatments, 3 countries (Hungary, the Netherlands, and Switzerland) showed a statistically significant difference (*P*<.05). Thus, in the remaining countries (n=20), there was no clear relationship between the reimbursement status of mHSPC, nmCRPC, and mCRPC treatments and their associated ESMO-MCBS scores (**Table 4**).

**Table 4.** Impact of Mean ESMO-MCBS Score on Reimbursement

**Country, n (% of Available Scores)**	**ESMO-MCBS 4-5**	**ESMO-MCBS <4**	**Difference (*P* Value)**
Austria			
Reimbursed	8 (100)	4 (80)	.3846
Not reimbursed	0 (0)	1 (20)	
Belgium			
Reimbursed	7 (88)	5 (100)	>.9999
Not reimbursed	1 (12)	0 (0)	
Bulgaria			
Reimbursed	7 (88)	4 (80)	>.9999
Not reimbursed	1 (12)	1 (20)	
Croatia			
Reimbursed	8 (100)	4 (80)	.3846
Not reimbursed	0 (0)	1 (20)	
Czech Republic			
Reimbursed	7 (88)	3 (60)	.5105
Not reimbursed	1 (12)	2 (40)	
Denmark			
Reimbursed	5 (63)	5 (100)	.2308
Not reimbursed	3 (37)	0 (0)	
Finland			
Reimbursed	8 (100)	4 (80)	.3846
Not reimbursed	0 (0)	1 (20)	
France			
Reimbursed	7 (88)	5 (100)	>.9999
Not reimbursed	1 (12)	0 (0)	
Germany			
Reimbursed	8 (100)	5 (100)	>.9999
Not reimbursed	0 (0)	0 (0)	
Greece			
Reimbursed	8 (100)	5 (100)	>.9999
Not reimbursed	0 (0)	0 (0)	
Hungary			
Reimbursed	6 (75)	0 (0)	.021
Not reimbursed	2 (25)	5 (100)	
Italy			
Reimbursed	6 (75)	3 (60)	>.9999
Not reimbursed	2 (25)	2 (40)	
The Netherlands			
Reimbursed	8 (100)	2 (40)	.035
Not reimbursed	0 (0)	3 (60)	
Norway			
Reimbursed	6 (75)	4 (80)	>.9999
Not reimbursed	2 (25)	1 (20)	
Poland			
Reimbursed	5 (63)	3 (60)	>.9999
Not reimbursed	3 (37)	2 (40)	
Portugal			
Reimbursed	6 (75)	3 (60)	>.9999
Not reimbursed	2 (25)	2 (40)	
Romania			
Reimbursed	5 (63)	3 (60)	>.9999
Not reimbursed	3 (37)	2 (40)	
Slovakia			
Reimbursed	4 (50)	1 (20)	.5649
Not reimbursed	4 (50)	4 (80)	
Slovenia			
Reimbursed	7 (88)	4 (80)	>.9999
Not reimbursed	1 (12)	1 (20)	
Spain			
Reimbursed	7 (88)	3 (60)	.5105
Not reimbursed	1 (12)	2 (40)	
Sweden			
Reimbursed	8 (100)	5 (100)	>.9999
Not reimbursed	0 (0)	0 (0)	
Switzerland			
Reimbursed	8 (100)	1 (20)	**.007**
Not reimbursed	0 (0)	4 (80)	
UK			
Reimbursed	6 (75)	2 (40)	.2929
Not reimbursed	2 (25)	3 (60)	

## DISCUSSION

In recent years, there has been an increase in the level of innovation among therapies for prostate cancer, which, in turn, challenges the capacity of HTA bodies to make dynamic, timely decisions, in keeping with the rate of EMA approval. As the global population continues to age, demand for anticancer treatments will continue to rise, requiring healthcare systems to make difficult decisions on where to best invest their funding. Country-specific decisions may also subject patients to pay out-of-pocket for treatment with no reimbursement, or with restricted reimbursement. With each country holding responsibility for assessing the benefit and cost-effectiveness of introducing new treatment options into their national healthcare systems, reimbursement decisions are therefore not unanimous across European countries. To mitigate these challenges, ESMO developed the MCBS to support the prioritization of treatments deemed to provide substantial therapeutic benefit to the patient. This review aimed to understand the reimbursement status and patient access to mHSPC, nmCRPC, and mCRPC treatments, and whether the ESMO-MCBS score had an impact on the reimbursement of therapies across European countries.

We found that the ESMO-MCBS scores of EMA-approved treatments ranged from 3 to 5, with the majority (9/13) considered to provide substantial benefit. Most of the countries included in this analysis provided positive reimbursement recommendations for those treatment regimens graded 4 and 5. However, despite these scores, decisions to reimburse were not consistent across indications or countries, and there was no clear relationship between the ESMO-MCBS scores and reimbursement status. These discrepancies highlight the variation across HTA bodies in Europe and the criteria used to assess and provide funding for anticancer treatments. Since 2020, 4 treatments have been approved by the EMA (ADT + apalutamide in mHSPC [2020], olaparib [2020], ADT + darolutamide [2020], and ADT + enzalutamide in mHSPC [2021]). Unlike ADT + apalutamide and ADT + enzalutamide, olaparib and ADT + darolutamide have not been approved for use in any other indications. To date, 11 of 23 and 14 of 23 countries, respectively, have not provided any form of reimbursement for these new anticancer treatments. This considerable delay by nearly half the countries included in this review highlights the inequity of patient access throughout Europe.

Two previously published studies have conducted an assessment of the impact of ESMO-MCBS grading on reimbursement in oncology drugs, specifically in Israel and Slovenia.^30,34^ Israeli reimbursement decisions were strongly aligned with ESMO-MCBS grading, with 58% of treatments that received a grade of 4 or 5 being reimbursed.^30^ Additionally, the ESMO-MCBS grading was found to assist with appropriate resource allocation, with 87% of low-benefit treatments (grade 0-2) not receiving approval.^30^ Alternatively, time to patient access following EMA authorization did not appear to be influenced by ESMO-MCBS grading in Slovenia.^34^ The median time to authorization across 53 anticancer drugs was 397 days (range, 98-615 days). When comparing treatments of substantial and nonsubstantial clinical benefit, authorization was granted in 393 vs 398 days, respectively.^34^ Similar studies have taken place to review the impact of other quality-appraisal methods on the reimbursement of anticancer treatments. In France, the Haute Authorité de Santé (HAS) grades novel treatments in respect to the therapeutic benefit from ASMR V (nonexistent benefit, lack of therapeutic progress) through to ASMR I, indicating major therapeutic progress^31^; these ASMR grades provide the basis for reimbursement. When comparing ASMR grades with ESMO-MCBS scores of new cancer drugs, there was a very weak correlation (r=0.27), with moderate agreement of benefit between the HAS ASMR and ESMO-MCBS scales.^36^ Although ESMO-MCBS can be an indicator of clinically meaningful benefit, it does not translate to the same assessment of value. Furthermore, cost-effectiveness remains a key consideration by HTA bodies in many countries; therefore, it is difficult to determine the direct association of the ESMO-MCBS score with overall reimbursement.

Unlike the previous studies, this review encompassed the reimbursement decisions across 23 European countries with a targeted focus on prostate cancer treatments and provided a region-level assessment of the reimbursement discrepancies that exist across Europe. It would be valuable to conduct similar analyses across different oncology indications to determine if the same discrepancies exist.

This study was limited to the publicly available material from HTA agencies; as a result, not all relevant information may have been captured and was subject to the transparency in reporting by the HTA body. The search of relevant documents took place between June and October 2022; therefore, further publications may have been released after the study’s conclusion. Despite including a vast range of European countries in this review, heterogeneity was apparent in the reporting across countries, making synthesis of available evidence challenging.

## CONCLUSIONS

The innovation and development of anticancer treatment has continued to evolve in recent years, providing multiple treatment options for patients with prostate cancer. Following the European Commission approval, country-specific HTA agencies are responsible for conducting their own assessment of new therapies to determine whether access should be granted to its population. These decisions vary across countries, with Greece being the only country included in this review to provide unrestricted reimbursement across all advanced prostate cancer drugs. The development of the ESMO-MCBS was intended to provide an evidence-based grading system to allow for prioritization of high-impact treatments for reimbursement. However, the association between the MCBS and reimbursement was unclear in the majority of countries included in this review. Continued development and understanding of ESMO-MCBS data in reimbursement decision could help decision-makers prioritize treatments that deliver the most value to patients and healthcare systems.

### Author Contributions

Conceptualization was carried out by G.B. and S.S.; methodology was developed and approved by G.B., S.S., S.M.P., E.E., D.S., and I.D.; validation of evidence was conducted by D.S. and I.D.; the formal analysis of results was conducted by G.B., S.M.P., and E.P.; data curation was carried out by S.M.P. and E.P.; writing of the original draft preparation was conducted by G.B., S.S., S.M.P., and E.P.; review and editing of manuscript was conducted by all authors; visualization was performed by G.B., S.S., S.M.P., and E.P.; the project was supervised by S.S.; project administration was carried out by G.B., S.M.P., and E.P.; and funding was acquired by G.B. and S.S.

### Disclosures

S.S., G.B., D.S., I.D., and E.P. are employees of Merck Sharp & Dohme LLC, a subsidiary of Merck & Co., Inc., Rahway, NJ, USA, and may own stocks and/or stock options in Merck & Co., Inc., Rahway, NJ, USA. S.M.P. is a former employee of MSD.

### Data Availability

Data are contained within the article or in the **Online** **Supplementary Material**.

## Supplementary Material

Online Supplementary Material
