# Supplementary material for: HTA and Reimbursement Status of Metastatic Hormone‑Sensitive Prostate Cancer, Non-Metastatic Castration-Resistant Prostate Cancer, and Metastatic Castration-Resistant Prostate Cancer Treatments in Europe: A Patient Access Landscape Review
Source: J Health Econ Outcomes Res. 2023 Jun 20;10(1):102–10. doi: 10.36469/001c.75208 (PMC10290826; doi:10.36469/001c.75208)
Supplement: Online Supplementary Material [file jheor_2023_10_1_75208_167458.pdf]

Journal of Health Economics and Outcomes Research

Online Supplementary Material

HTA and Reimbursement Status of Metastatic Hormone-Sensitive Prostate Cancer, Nonmetastatic Castration-Resistant Prostate Cancer, and Metastatic Castration-Resistant Prostate Cancer Treatments in Europe: A Patient Access Landscape Review. *JHEOR*. 2023;10(1):102-110. doi:10.36469/jheor.2023.75208

Supplementary Appendix: Country-Specific Restrictions of Advanced Prostate Cancer Treatments

This supplementary material has been provided by the authors to give readers additional information about their work.

This is an open-access article distributed under the terms of the Creative Commons Attribution 4.0 International License (CCBY-4.0). View this license's legal deed at http://creativecommons.org/licenses/by/4.0 and legal code at http://creativecommons.org/licenses/by/4.0/legalcode for more information.

SUPPLEMENTARY APPENDIX

Country-Specific Restrictions of Advanced Prostate Cancer Treatments

Country	Description of Restrictions	
Austria [20-22]	**Metastatic hormone-sensitive prostate cancer**	
	ADT + abiraterone	Yellow box (reimbursed with prior validation of chief-doctor)
	ADT + enzalutamide	Yellow box (reimbursed with prior validation of chief-doctor)
	ADT + apalutamide	Yellow box (reimbursed with prior validation of chief-doctor)
	**Metastatic castration-resistant prostate cancer (1st-line)**	
	Abiraterone	Yellow box (reimbursed with prior validation of chief-doctor)
	Enzalutamide	Yellow box (reimbursed with prior validation of chief-doctor)
	**Metastatic castration-resistant prostate cancer (2nd line/post chemo)**	
	Abiraterone	Yellow box (reimbursed with prior validation of chief-doctor)
	Enzalutamide	Yellow box (reimbursed with prior validation of chief-doctor)
	**Nonmetastatic castration-resistant prostate cancer**	
	ADT + apalutamide	Yellow box (reimbursed with prior validation of chief-doctor)
	ADT + darolutamide	Yellow box (reimbursed with prior validation of chief-doctor)
Belgium [23]	**Metastatic hormone-sensitive prostate cancer**	
	ADT + abiraterone	In association with prednisone or prednisolone. The patients respond to 2 following criteria: has a polymetastatic disease with multiple lesions and/or 1visceral metastasis and/or a Gleason score of ≥8; is not eligible to a treatment with docetaxel because of objective reasons.
	ADT + apalutamide	In case of ineligibility to docetaxel for objective reasons, physician is asked to stop the treatment in case of disease progression as demonstrated by ≥2 of following signs: PSA of more than 2 ng/mL or 25% more than the minimal level recorded during the treatment and confirmed ≥3 weeks later; progression of bone lesions; progression of soft tissue lesions according to RECIST; appearance of one or several soft tissue lesions.
	**Metastatic castration-resistant prostate cancer (1st line)**	
	Olaparib	In monotherapy in tumors with BRCA 1/2 (germinal or somatic) mutation after progression under a new hormonal treatment (such as abiraterone, enzalutamide, apalutamide, darolutamide or similar products). The patient responds to the following criteria: Testosterone castration concentrations <50 ng/dL or <1,7 nmol/L; deleterious mutation confirmed or suspected of BRCA1/2 gene; previously treated with a new hormonal agent; presenting a progression of the disease after a new hormonal agent: 3 consecutive increases of PSA; progression of bone mets; progression of soft tissue mets /appearance of one or several visceral or soft tissue mets; ineligibility to docetaxel because of: progression, intolerance or contra-indication.

Country	Description of Restrictions	
	Abiraterone	With prednisone or prednisolone in asymptomatic or with little symptoms after failing an androgenic suppression and for which chemotherapy is not yet indicated. The conditions are the following: serum castration testosterone concentrations of <50 ng/dL or <1.7 nmol/L; not yet treated with abiraterone for hormone sensitive prostate cancer in newly diagnosed patients with metastasis; Brief Pain Inventory - Short Form (BPI-SF) pain score of 0-3 for the most intense pain during previous 24 hours who present at least one of the following signs: 3 successive increases of serum PSA (at least one >2 ng/mL and representing an increase of ≥50% vs nadir PSA; progression of bone mets progression of soft tissue lesions; appearance of one or several soft tissue or solid organ mets; not yet eligible to docetaxel.
	Enzalutamide	In asymptomatic or minimally symptomatic patients when chemotherapy not yet indicated. The patient needs to respond to following criteria: testosterone castration levels of <50 ng/dL or <1.7 nmol/L; pain score of 0-3 in the BPI-SF (most intense pain in the last 24 hours); presents at least one of the following signs of disease progression: 3 consecutive increases of PSA with one at least >2 ng/mL and an increase of ≥50% vs PSA nadir; progression of bone mets; progressions of soft tissue lesions according to RECIST; appearance of visceral or soft tissue mets; is not yet eligible for starting a treatment with docetaxel (PSA doubling time of >6 months); keeps being treated with medical castration (if not surgical castration).
	**Metastatic castration-resistant prostate cancer (2nd line/post chemo)**	
	Abiraterone	In association with prednisone or prednisolone progressing during or after a treatment with docetaxel. The patient responds to following criteria: serum castration testosterone <50 ng/mL or <1.7 nmol/L not yet treated with abiraterone in 1st line (for a metastatic prostate cancer resistant to castration in men with no or little symptoms after failure of a androgenic suppression and when chemotherapy not yet needed/has received at least 3 cycles of docetaxel; not eligible to a 2nd treatment with docetaxel because of either a progressive disease under docetaxel, progressive disease <5 months after last dose of docetaxel in 1st line, either intolerance to docetaxel demonstrated during a 1st line treatment; presents at least one of the following signs of progression of disease: 3 PSA level increase (with at least one concentration of >2 ng/mL and representing at least 50% increase vs nadir PSA; progression of bone metastasis; progression of soft tissues lesions; new organ metastasis or of soft tissues.
	Cabazitaxel	In association to prednisone progressing during or after docetaxel treatment with the following criteria: castration testosterone <50 ng/dL or 1.7 nmol/L; has been treated with flutamide or bicalutamide or by secondary hormonal manipulation; has received ≥3 cycles of docetaxel; is not eligible to a 2nd treatment with docetaxel because of the following reasons: disease progression during a 1st-line treatment with docetaxel; progression of the disease within 5 months post-docetaxel treatment; docetaxel intolerance during docetaxel treatment. The patient has an ECOG 0 or 1 and presents at least one of the following signs: 3 consecutive PSA increase with ≥2 measures of >2 ng/mL and ≥50% increase vs. PSA nadir; progression of bone

Country	Description of Restrictions	
		lesions; progression of soft tissue according to RECIST/appearance of one or several visceral mets.
	Enzalutamide	Progression under docetaxel with following criteria: testosterone serum concentration of <50 ng/dL or <1.7 nmol/L: enzalutamide naïve in 1st line in asymptomatic or little symptoms patients after failure of androgenic treatment and when chemotherapy not yet indicated; receive ≥3 cycles of docetaxel, except in case of intolerance during 1st line treatment; not eligible to a 2nd docetaxel treatment because of one of the following criteria: disease progression under 1st line docetaxel (≥3 cycles); disease progression <5 months after last docetaxel administration in 1st line; intolerance to docetaxel; has at least one of the following signs of progression of the disease: 3 consecutive PSA increases, progression of bone mets, progression of soft tissue lesions, appearance of one or several visceral mets or soft tissue.
	Olaparib	In monotherapy in tumors with BRCA 1/2 (germinal or somatic) after progression under a new hormonal treatment (such as abiraterone, enzalutamide, apalutamide, darolutamide or similar products). The patient responds to the following criteria: Testosterone serum castration concentration <50 ng/dL or <1.7 nmol/L; deleterious mutation confirmed or suspected of BRCA 1/2 gene (germinal and/or somatic); has been previously treated with a new hormonal agent; presents at least one sign of progression after a previous treatment with a novel antihormonal treatment: 3 consecutive PSA increases, progression of bone mets, progression of soft tissue lesions according to RECIST; appearance of one or several visceral or soft tissue lesions. In addition, the patient is not a candidate for docetaxel for one of the following reasons: progression of the disease after a previous docetaxel treatment, intolerance, contra-indication. The patient is also not eligible to cabazitaxel because of a contraindication. The patient will pursue the drug castration.
	**Nonmetastatic castration-resistant prostate cancer**	
	ADT + apalutamide	In high-risk patients with following criteria: castration testosterone serum concentration <50 ng/dL or <1.7 nmol/L; PSA increase and 2 ng/mL more than the nadir and which represents an increase of at least 25% vs nadir PSA, confirmed by a 2nd value; PSA doubling time ≤10 months; no proof of bone mets on bone scan and CT scan/NMR.
	ADT + darolutamide	In high-risk patients with following criteria: castration testosterone serum concentration <50 ng/dL or <1,7 nmol/L; PSA increase and 2 ng/mL more than the nadir and which represents an increase of at least 25% vs nadir PSA, confirmed by a 2nd value; PSA doubling time of ≥10 months; no proof of mets on bone scan or CT scan/NMR.
	ADT + enzalutamide	In high-risk patients with following criteria: castration testosterone serum concentration <50 ng/dL or <1,7 nmol/L; PSA increase and 2 ng/ml more than the nadir and which represents an increase of at least 25% vs nadir PSA, confirmed by a 2nd value; PSA doubling time ≤10 months; no evidence of mets on bone scan and CT scan/NMR. Lymphatic mets under the aortic split are allowed.
	**Metastatic castration-resistant prostate cancer with symptomatic bone metastases**	

Country	Description of Restrictions	
	Radium Ra 223 dichloride	The patient must meet each of the following criteria at the initiation of treatment with XOFIGO: serum castration testosterone level <50 ng/dL or <1.7 nmol/L after bilateral orchiectomy or under androgen deprivation therapy; multiple bone metastases (≥2 hot spots) on bone scan or other medical imaging; use of non-opioid or opioid analgesics for cancer-related bone pain on a regular basis or external radiation therapy for bone pain in the past 12 weeks or presence of neurological symptoms; absence or presence of malignant lymphadenopathy with largest lymph nodes <3 cm (diameter short axis); absence of visceral metastases confirmed by medical imaging of the abdomen and small pelvis (CT or MRI) and thorax (x-ray or CT) not older than 8 weeks; has ≥1 of the following signs of disease progression: progression of bone lesions (appearance of additional bone lesions on bone scan); 3 consecutive increases in serum PSA (with an interval of ≥7 days) including at least 2 with a serum PSA >2 ng/mL and representing an increase of at least 50% compared to the nadir PSA; progression of bone pain (eg, increased use of non-opioid or opioid analgesics or bone pain after external radiation therapy in the last 12 weeks).<br><br>If the patient has not yet been treated with docetaxel for his prostate cancer or is not eligible to start treatment with docetaxel: the patient has a time rate doubling of PSA for more than 6 months (calculated according to Arlen et al. J'Urol. 2008 June; 179 (6): 2181–2186) or is not eligible to start treatment with docetaxel for other reasons.<br><br>If the patient has already been treated with docetaxel for his prostate cancer: has already received at least 3 cycles of docetaxel for the said indication (at a dose of ≥225 mg/ m2), unless an intolerance has been demonstrated during 1st-line treatment with docetaxel; is not eligible for a 2nd treatment with docetaxel due to one of the following: has progressed during 1st-line treatment with docetaxel (at least 3 cycles); showed progression <5 months after last 1st-line docetaxel administration; showed demonstrable intolerance during 1st-line treatment with docetaxel. The number of reimbursable packaging will take into account a dosage of XOFIGO corresponding to 55 kBq per kg of body weight (maximum 1 vial is reimbursable per administration) administered by injection every 4 weeks, with a maximum of 6 shots. If the patient requires 2 vials per administration, reimbursement for a maximum of 1 vial per administration will be considered. The 2nd vial per administration will in this case be provided free of charge by the firm (with a maximum of 6 free vials for the total treatment of the patient).
Croatia [24]	**Metastatic hormone-sensitive prostate cancer**	
	ADT + abiraterone	For the treatment of patients with castration-resistant metastatic prostate cancer who are asymptomatic or have mild symptoms after failure of androgen deprivation treatment and in whom chemotherapy is not yet clinically indicated. The drug is administered in combination with prednisone or prednisolone. Three cycles of treatment are approved, after which the effect of therapy and tolerability of treatment is assessed on the basis of diagnostic processing. Treatment is carried out until the disease progresses. Progression is considered to be a significant worsening of the disease

Country	Description of Restrictions	
		based on the assessment of clinical progression and at least 1 of 2 additional criteria (PSA value and/or radiological progression). Treatment is approved by the Hospital Medicines Committee.<br>For the 2nd-line treatment of patients with castration-resistant metastatic prostate cancer whose disease has progressed during or after a docetaxel-based chemotherapy protocol at a cumulative/total dose of ≥225 mg/m^2. The drug is administered in combination with prednisone or prednisolone. Three cycles of treatment are approved, after which the effect of therapy and tolerability of treatment is assessed on the basis of diagnostic processing. Treatment is carried out until the disease progresses. Progression is considered to be a significant worsening of the disease based on the assessment of clinical progression and at least 1 of 2 additional criteria (PSA value and/or radiological progression). Treatment is approved by the Hospital Medicines Committee.<br><br>For the treatment of adult men with newly diagnosed high-risk hormone-sensitive metastatic prostate cancer in combination with androgen deprivation therapy who are not candidates for chemotherapy or who have not responded to or are intolerant to docetaxel therapy. Three cycles of treatment are approved, after which the effect of therapy and tolerability of treatment is assessed on the basis of diagnostic processing. Treatment is carried out until the disease progresses. Progression is considered to be a significant worsening of the disease based on the assessment of clinical progression and at least 1 of 2 additional criteria (PSA value and/or radiological progression). Treatment is approved by the Hospital Medicines Committee.
	ADT + enzalutamide	For the treatment of patients with castration-resistant metastatic prostate cancer who are asymptomatic or have mild symptoms after failure of androgen deprivation treatment and in whom chemotherapy is not yet clinically indicated. Three cycles of treatment are approved, after which the effect of therapy and tolerability of treatment is assessed on the basis of diagnostic processing. Treatment is carried out until the disease progresses. Progression is considered to be a significant worsening of the disease based on the assessment of clinical progression and at least 1 of 2 additional criteria (PSA value and/or radiological progression). Treatment is approved by the Hospital Medicines Committee.<br><br>For the 2nd-line treatment of patients with castration-resistant metastatic prostate cancer whose disease has progressed during or after a docetaxel-based chemotherapy protocol at a cumulative/total dose of at least 225 mg/m^2. Three cycles of treatment are approved, after which the effect of therapy and tolerability of treatment is assessed on the basis of diagnostic processing. Treatment is carried out until the disease progresses. Progression is considered to be a significant worsening of the disease based on the assessment of clinical progression and at least 1 of 2 additional criteria (PSA value and/or radiological progression). Treatment is approved by the Hospital Medicines Committee.<br>For the treatment of patients with nonmetastatic, castration-resistant prostate cancer (nmCRPC) who meet the criteria of high-risk

Country	Description of Restrictions	
		definition or in whom the doubling time of PSA ≤ 0 months (PSA-DT ≤10 months), ECOG status 0-1. Clinical and diagnostic processing is required every 3 months to assess the effect of therapy and tolerability of treatment. Treatment is carried out until disease progression, which is considered a significant worsening of the disease based on the assessment of clinical progression and at least 1 of 2 additional criteria (PSA value and/or radiological progression). Treatment is approved by the Hospital Medicines Committee.<br><br>For the treatment of adult men with hormone-sensitive metastatic prostate cancer in combination with androgen deprivation therapy. Three cycles of treatment are approved, after which the effect of therapy and tolerability of treatment is assessed on the basis of diagnostic processing. Treatment is carried out until the disease progresses. Progression is considered to be a significant worsening of the disease based on the assessment of clinical progression and at least 1 of 2 additional criteria (PSA value and/or radiological progression). Treatment is approved by the Hospital Medicines Committee.
	ADT + apalutamide	For the treatment of patients with non-metastatic, castration-resistant prostate cancer (nmCRPC) who meet the criteria of high-risk definition or in whom the doubling time of PSA ≤ 10 months (PSA-DT ≤10 months), ECOG status 0-1. Clinical and diagnostic processing is required every 3 months to assess the effect of therapy and tolerability of treatment. Treatment is carried out until disease progression, which is considered a significant worsening of the disease based on the assessment of clinical progression and at least 1 of 2 additional criteria (PSA value and/or radiological progression). Treatment is approved by the Hospital Medicines Committee.<br><br>For the treatment of adult men with hormone-sensitive metastatic prostate cancer in combination with androgen deprivation therapy. Three cycles of treatment are approved, after which the effect of therapy and tolerability of treatment is assessed on the basis of diagnostic processing. Treatment is carried out until the disease progresses. Progression is considered to be a significant worsening of the disease based on the assessment of clinical progression and at least 1 of 2 additional criteria (PSA value and/or radiological progression). Treatment is approved by the Hospital Medicines Committee.
	**Metastatic castration-resistant prostate cancer (1st line)**	
	Abiraterone	For the treatment of patients with castration-resistant metastatic prostate cancer who are asymptomatic or have mild symptoms after failure of androgen deprivation treatment and in whom chemotherapy is not yet clinically indicated. The drug is administered in combination with prednisone or prednisolone. Three cycles of treatment are approved, after which the effect of therapy and tolerability of treatment is assessed on the basis of diagnostic processing. Treatment is carried out until the disease progresses. Progression is considered to be a significant worsening of the disease based on the assessment of clinical progression and at least 1 of 2 additional criteria (PSA value and/or radiological progression). Treatment is approved by the Hospital Medicines Committee.

Country	Description of Restrictions	
		For the 2nd-line treatment of patients with castration-resistant metastatic prostate cancer whose disease has progressed during or after a docetaxel-based chemotherapy protocol at a cumulative/total dose of ≥225 mg/m^2. The drug is administered in combination with prednisone or prednisolone. Three cycles of treatment are approved, after which the effect of therapy and tolerability of treatment is assessed on the basis of diagnostic processing. Treatment is carried out until the disease progresses. Progression is considered to be a significant worsening of the disease based on the assessment of clinical progression and at least 1 of 2 additional criteria (PSA value and/or radiological progression). Treatment is approved by the Hospital Medicines Committee.<br><br>For the treatment of adult men with newly diagnosed high-risk hormone-sensitive metastatic prostate cancer in combination with androgen deprivation therapy who are not candidates for chemotherapy or who have not responded to or are intolerant to docetaxel therapy. Three cycles of treatment are approved, after which the effect of therapy and tolerability of treatment is assessed on the basis of diagnostic processing. Treatment is carried out until the disease progresses. Progression is considered to be a significant worsening of the disease based on the assessment of clinical progression and at least 1 of 2 additional criteria (PSA value and/or radiological progression). Treatment is approved by the Hospital Medicines Committee.
	Enzalutamide	For the treatment of patients with castration-resistant metastatic prostate cancer who are asymptomatic or have mild symptoms after failure of androgen deprivation treatment and in whom chemotherapy is not yet clinically indicated. Three cycles of treatment are approved, after which the effect of therapy and tolerability of treatment is assessed on the basis of diagnostic processing. Treatment is carried out until the disease progresses. Progression is considered to be a significant worsening of the disease based on the assessment of clinical progression and at least 1 of 2 additional criteria (PSA value and/or radiological progression). Treatment is approved by the Hospital Medicines Committee.<br><br>For the 2nd-line treatment of patients with castration-resistant metastatic prostate cancer whose disease has progressed during or after a docetaxel-based chemotherapy protocol at a cumulative/total dose of at least 225 mg/m^2. Three cycles of treatment are approved, after which the effect of therapy and tolerability of treatment is assessed on the basis of diagnostic processing. Treatment is carried out until the disease progresses. Progression is considered to be a significant worsening of the disease based on the assessment of clinical progression and at least 1 of 2 additional criteria (PSA value and/or radiological progression). Treatment is approved by the Hospital Medicines Committee.<br>For the treatment of patients with non-metastatic, castration-resistant prostate cancer (nmCRPC) who meet the criteria of high-risk definition or in whom the doubling time of PSA ≤ 10 months (PSA-DT ≤10 months), ECOG status 0-1. Clinical and diagnostic

Country	Description of Restrictions	
		processing is required every 3 months to assess the effect of therapy and tolerability of treatment. Treatment is carried out until disease progression, which is considered a significant worsening of the disease based on the assessment of clinical progression and at least 1 of 2 additional criteria (PSA value and/or radiological progression). Treatment is approved by the Hospital Medicines Committee.<br><br>For the treatment of adult men with hormone-sensitive metastatic prostate cancer in combination with androgen deprivation therapy. Three cycles of treatment are approved, after which the effect of therapy and tolerability of treatment is assessed on the basis of diagnostic processing. Treatment is carried out until the disease progresses. Progression is considered to be a significant worsening of the disease based on the assessment of clinical progression and at least 1 of 2 additional criteria (PSA value and/or radiological progression). Treatment is approved by the Hospital Medicines Committee.
	**Metastatic castration-resistant prostate cancer (2nd line/post chemo)**	
	Abiraterone	For the treatment of patients with castration-resistant metastatic prostate cancer who are asymptomatic or have mild symptoms after failure of androgen deprivation treatment and in whom chemotherapy is not yet clinically indicated. The drug is administered in combination with prednisone or prednisolone. Three cycles of treatment are approved, after which the effect of therapy and tolerability of treatment is assessed on the basis of diagnostic processing. Treatment is carried out until the disease progresses. Progression is considered to be a significant worsening of the disease based on the assessment of clinical progression and at least 1 of 2 additional criteria (PSA value and/or radiological progression). Treatment is approved by the Hospital Medicines Committee.<br>For the 2nd-line treatment of patients with castration-resistant metastatic prostate cancer whose disease has progressed during or after a docetaxel-based chemotherapy protocol at a cumulative/total dose of at least 225 mg/m^2. The drug is administered in combination with prednisone or prednisolone. Three cycles of treatment are approved, after which the effect of therapy and tolerability of treatment is assessed on the basis of diagnostic processing. Treatment is carried out until the disease progresses. Progression is considered to be a significant worsening of the disease based on the assessment of clinical progression and at least 1 of 2 additional criteria (PSA value and/or radiological progression). Treatment is approved by the Hospital Medicines Committee.<br><br>For the treatment of adult men with newly diagnosed high-risk hormone-sensitive metastatic prostate cancer in combination with androgen deprivation therapy who are not candidates for chemotherapy or who have not responded to or are intolerant to docetaxel therapy. Three cycles of treatment are approved, after

Country	Description of Restrictions	
		which the effect of therapy and tolerability of treatment is assessed on the basis of diagnostic processing. Treatment is carried out until the disease progresses. Progression is considered to be a significant worsening of the disease based on the assessment of clinical progression and at least 1 of 2 additional criteria (PSA value and/or radiological progression). Treatment is approved by the Hospital Medicines Committee.
	Cabazitaxel	For 2nd-line treatment of patients with castration-resistant metastatic prostate cancer (in patients who have progressed during or after docetaxel-based chemotherapy protocol, at a cumulative/total dose of ≥225 mg/m^2). The drug is used in combination with prednisone or prednisolone, in patients who have a physical status of 0 or 1 according to ECOG. Three cycles of treatment are approved, after which the effect of therapy and tolerability of treatment is assessed on the basis of diagnostic processing. Treatment is carried out until the disease progresses. Progression is considered to be a significant worsening of the disease based on the assessment of clinical progression and at least 1 of 2 additional criteria (PSA value and/or radiological progression). Treatment is approved by the Hospital Medicines Committee.
	Enzalutamide	For the treatment of patients with castration-resistant metastatic prostate cancer who are asymptomatic or have mild symptoms after failure of androgen deprivation treatment and in whom chemotherapy is not yet clinically indicated. Three cycles of treatment are approved, after which the effect of therapy and tolerability of treatment is assessed on the basis of diagnostic processing. Treatment is carried out until the disease progresses. Progression is considered to be a significant worsening of the disease based on the assessment of clinical progression and at least 1 of 2 additional criteria (PSA value and/or radiological progression). Treatment is approved by the Hospital Medicines Committee.<br>For the 2nd-line treatment of patients with castration-resistant metastatic prostate cancer whose disease has progressed during or after a docetaxel-based chemotherapy protocol at a cumulative/total dose of at least 225 mg/m^2. Three cycles of treatment are approved, after which the effect of therapy and tolerability of treatment is assessed on the basis of diagnostic processing. Treatment is carried out until the disease progresses. Progression is considered to be a significant worsening of the disease based on the assessment of clinical progression and at least 1 of 2 additional criteria (PSA value and/or radiological progression). Treatment is approved by the Hospital Medicines Committee.<br><br>For the treatment of patients with non-metastatic, castration-resistant prostate cancer (nmCRPC) who meet the criteria of high-risk definition or in whom the doubling time of PSA ≤10 months (PSA-DT ≤10 months), ECOG status 0-1. Clinical and diagnostic processing is required every 3 months to assess the effect of therapy and tolerability of treatment. Treatment is carried out until disease progression, which is considered a significant worsening of the disease based on the assessment of clinical progression and at least 1 of 2 additional criteria (PSA value and/or radiological progression). Treatment is approved by the Hospital Medicines Committee.

Country	Description of Restrictions	
		For the treatment of adult men with hormone-sensitive metastatic prostate cancer in combination with androgen deprivation therapy. Three cycles of treatment are approved, after which the effect of therapy and tolerability of treatment is assessed on the basis of diagnostic processing. Treatment is carried out until the disease progresses. Progression is considered to be a significant worsening of the disease based on the assessment of clinical progression and at least 1 of 2 additional criteria (PSA value and/or radiological progression). Treatment is approved by the Hospital Medicines Committee.
	**Non-metastatic castration-resistant prostate cancer**	
	ADT + apalutamide	For the treatment of patients with non-metastatic, castration-resistant prostate cancer (nmCRPC) who meet the criteria of high-risk definition or in whom the doubling time of PSA ≤10 months (PSA-DT ≤10 months), ECOG status 0-1. Clinical and diagnostic processing is required every 3 months to assess the effect of therapy and tolerability of treatment. Treatment is carried out until disease progression, which is considered a significant worsening of the disease based on the assessment of clinical progression and at least 1 of 2 additional criteria (PSA value and/or radiological progression). Treatment is approved by the Hospital Medicines Committee.<br><br>For the treatment of adult men with hormone-sensitive metastatic prostate cancer in combination with androgen deprivation therapy. Three cycles of treatment are approved, after which the effect of therapy and tolerability of treatment is assessed on the basis of diagnostic processing. Treatment is carried out until the disease progresses. Progression is considered to be a significant worsening of the disease based on the assessment of clinical progression and at least 1 of 2 additional criteria (PSA value and/or radiological progression). Treatment is approved by the Hospital Medicines Committee.
	ADT + darolutamide	For the treatment of patients with nonmetastatic, castration-resistant prostate cancer (nmCRPC) who meet the criteria for the definition of high risk, ie, in whom the doubling time of PSA ≤10 months (PSA-DT ≤10 months), ECOG status 0-1. Clinical and diagnostic processing is required every 3 months to assess the effect of therapy and tolerability of treatment. Treatment is carried out until disease progression, which is considered a significant worsening of the disease based on the assessment of clinical progression and at least 1 of 2 additional criteria (PSA value and/or radiological progression). Treatment is approved by the Hospital Medicines Committee.

Country	Description of Restrictions	
	ADT + enzalutamide	For the treatment of patients with castration-resistant metastatic prostate cancer who are asymptomatic or have mild symptoms after failure of androgen deprivation treatment and in whom chemotherapy is not yet clinically indicated. Three cycles of treatment are approved, after which the effect of therapy and tolerability of treatment is assessed on the basis of diagnostic processing. Treatment is carried out until the disease progresses. Progression is considered to be a significant worsening of the disease based on the assessment of clinical progression and at least 1 of 2 additional criteria (PSA value and/or radiological progression). Treatment is approved by the Hospital Medicines Committee.<br><br>For the 2nd-line treatment of patients with castration-resistant metastatic prostate cancer whose disease has progressed during or after a docetaxel-based chemotherapy protocol at a cumulative/total dose of at least 225 mg/m^2. Three cycles of treatment are approved, after which the effect of therapy and tolerability of treatment is assessed on the basis of diagnostic processing. Treatment is carried out until the disease progresses. Progression is considered to be a significant worsening of the disease based on the assessment of clinical progression and at least 1 of 2 additional criteria (PSA value and/or radiological progression). Treatment is approved by the Hospital Medicines Committee.<br><br>For the treatment of patients with non-metastatic, castration-resistant prostate cancer (nmCRPC) who meet the criteria of high-risk definition or in whom the doubling time of PSA ≤10 months (PSA-DT ≤10 months), ECOG status 0-1. Clinical and diagnostic processing is required every 3 months to assess the effect of therapy and tolerability of treatment. Treatment is carried out until disease progression, which is considered a significant worsening of the disease based on the assessment of clinical progression and at least 1 of 2 additional criteria (PSA value and/or radiological progression). Treatment is approved by the Hospital Medicines Committee.<br><br>For the treatment of adult men with hormone-sensitive metastatic prostate cancer in combination with androgen deprivation therapy. Three cycles of treatment are approved, after which the effect of therapy and tolerability of treatment is assessed on the basis of diagnostic processing. Treatment is carried out until the disease progresses. Progression is considered to be a significant worsening of the disease based on the assessment of clinical progression and at least 1 of 2 additional criteria (PSA value and/or radiological progression). Treatment is approved by the Hospital Medicines Committee.

Country	Description of Restrictions	
	**Metastatic castration-resistant prostate cancer with symptomatic bone metastases**	
	Radium Ra 223 dichloride	For the treatment of adult patients with castration-resistant metastatic prostate cancer in monotherapy or in combination with a luteinizing hormone-releasing hormone (LHRH) analogue, with symptomatic bone metastases and no known visceral metastases, which is in progression after at least two previous lines of systemic therapy therapies for metastatic castration-resistant prostate cancer (except LHRH analogues) with ECOG status 0-2. Six treatment cycles are approved. The treatment is approved by the Clinical Hospital Committee for Medicines with the prior opinion of a multidisciplinary team.<br><br>In patients with castration-resistant metastatic prostate cancer in monotherapy or in combination with an LHRH analog, with symptomatic bone metastases and without known visceral metastases, who are not suitable for treatment with available systemic therapy (chemotherapy protocol based on docetaxel/cabazitaxel or new hormonal therapy abiraterone acetate/enzalutamide protocol) with ECOG status 0-2. Six treatment cycles are approved. The treatment is approved by the Clinical Hospital Committee for Medicines with the prior opinion of a multidisciplinary team.
Czech Republic [25]	**Metastatic hormone-sensitive prostate cancer**	
	ADT + abiraterone	High-risk patients, ECOG 0-1, therapy reimbursed until progression, in combination with prednisone/prednisolone.
	ADT + apalutamide	In combination with ADT, ECOG 0-1, reimbursed until progression.
	**Metastatic castration-resistant prostate cancer (1st line)**	
	Abiraterone	Asymptomatic patients, or with mild symptoms, ECOG 0-1, without cancer pain symptoms (BPI 0-1), therapy reimbursed until progression, in combination with prednisone/prednisolone.
	Enzalutamide	Asymptomatic patients, or mild symptoms, ECOG 0-1, therapy reimbursed until progression.
	**Metastatic castration-resistant prostate cancer (2nd line/post chemo)**	
	Abiraterone	Patients with progression on docetaxel, ECOG 0-2, therapy reimbursed until progression, in combination with prednisone/prednisolone.
	Cabazitaxel	Patients with progression on docetaxel, ECOG 0-2, in combination with prednisone/prednisolone, therapy reimbursed until progression, maximal length of treatment 10 cycles, not reimbursed for patients with peripheral neuropathy or stomatitis grade 2.
	Enzalutamide	Patients with progression on docetaxel, ECOG 0-2, therapy reimbursed until progression.
	**Non-metastatic castration-resistant prostate cancer**	
	ADT + apalutamide	Patients with high risk of metastasis, ECOG 0-1, in combination with ADT, treatment reimbursed until progression.
	ADT + darolutamide	Patients with high risk of metastasis, ECOG 0-1, in combination with ADT, treatment reimbursed until progression.

Country	Description of Restrictions	
	**Metastatic castration-resistant prostate cancer with symptomatic bone metastases**	
	Radium Ra 223 dichloride	Patients without visceral metastasis or malignant lymphadenopathy bigger than 3 cm, after docetaxel treatment (or if docetaxel can´t be used), ECOG 0-2.
Denmark[26]	**Metastatic castration-resistant prostate cancer (1st line)**	
	Abiraterone	Mandatory to use drug with lowest cost.
	Enzalutamide	Mandatory to use drug with lowest cost.
	**Metastatic castration-resistant prostate cancer (2nd line/post chemo)**	
	Abiraterone	Mandatory to use drug with lowest cost and no sequential use of Abiraterone and Enzalutamide.
	Cabazitaxel	Mandatory to use drug with lowest cost.
	Enzalutamide	Mandatory to use drug with lowest cost and no sequential use of Abiraterone and Enzalutamide.
	Olaparib	Use post Cabazitaxel and docetaxel.
	**Non-metastatic castration-resistant prostate cancer**	
	ADT + apalutamide	Mandatory to use drug with lowest cost.
	ADT + darolutamide	Mandatory to use drug with lowest cost.
	ADT + enzalutamide	Mandatory to use drug with lowest cost.
	**Metastatic castration-resistant prostate cancer with symptomatic bone metastases**	
	Radium Ra 223 dichloride	Patients with symptomatic bone metastases without visceral or lymph node (>3 cm) metastases may be offered treatment with radium-223 after ≥2 lines of systemic therapy for mCRPC or if they are unfit for other mCRPC therapy (A).
Finland [27]	**Metastatic hormone-sensitive prostate cancer**	
	ADT + abiraterone	Abiraterone is subject to special reimbursement. The right to special compensation is granted on the basis of the B-statement issued from the unit treating cancer diseases in specialized medical care for the treatment of metastatic castration-resistant prostate cancer in adults whose disease has progressed during or after docetaxel-based chemotherapy, or who are asymptomatic or mildly symptomatic after failure of androgen deprivation therapy and for whom chemotherapy is not yet necessary.<br><br>In addition to information on the disease and its previous treatment and treatment outcomes, the statement shall include a treatment plan in accordance with good medical practice. The right to reimbursement shall be granted for a fixed period of time as required by the appropriate management plan, but for a maximum of 3 years at a time.
	ADT + enzalutamide	Enzalutamide is subject to limited special reimbursement in the treatment of prostate cancer under the following conditions. The right to special reimbursement is granted on the basis of the B-statement on the unit treating cancer in specialized medical care to adults with prostate cancer for the treatment of metastatic castration-resistant disease when: The doubling period of PSA (prostate-specific antigen) is up to 10 months (high risk of

Country	Description of Restrictions	
		developing metastatic disease). for the treatment of metastatic castration-resistant disease when: the disease has progressed during or after docetaxel-based chemotherapy, or the patient is asymptomatic or mildly symptomatic after failure of androgen deprivation therapy and chemotherapy is not yet necessary. In addition to information on the disease and its previous treatment and treatment outcomes, the statement shall include a treatment plan in accordance with good medical practice.
	ADT + apalutamide	Reimbursement requested and granted based on clinicians written statement.
	**Metastatic castration-resistant prostate cancer (1st line)**	
	Abiraterone	Reimbursement requested and granted based on clinicians written statement.
	Enzalutamide	Reimbursement requested and granted based on clinicians written statement.
	**Metastatic castration-resistant prostate cancer (2nd line/post chemo)**	
	Abiraterone	Reimbursement requested and granted based on clinicians written statement.
	Enzalutamide	Reimbursement requested and granted based on clinicians written statement.
	**Non-metastatic castration-resistant prostate cancer**	
	ADT + apalutamide	When the cancer has metastasized to other parts of the body and drug therapy or surgery to reduce testosterone levels is still effective (ie, hormone-sensitive prostate cancer) or when the cancer has not metastasized to other parts of the body and drug therapy or surgery to reduce testosterone levels is no longer effective (ie, so-called castration-resistant prostate cancer).
	ADT + darolutamide	When the cancer has not spread to other parts of the body and when drug therapy or surgery that reduces testosterone levels is no longer effective (ie, so-called castration-resistant prostate cancer).
	ADT + enzalutamide	Enzalutamide is subject to limited special reimbursement in the treatment of prostate cancer under the following conditions. The right to special reimbursement is granted on the basis of the B-statement on the unit treating cancer in specialized medical care to adults with prostate cancer for the treatment of metastatic castration-resistant disease when: The doubling period of PSA (prostate-specific antigen) is up to 10 months (high risk of developing metastatic disease). for the treatment of metastatic castration-resistant disease when: the disease has progressed during or after docetaxel-based chemotherapy, or the patient is asymptomatic or mildly symptomatic after failure of androgen deprivation therapy and chemotherapy is not yet necessary. In addition to information on the disease and its previous treatment and treatment outcomes, the statement shall include a treatment plan in accordance with good medical practice.
Germany [28]	**Metastatic hormone-sensitive prostate cancer**	
	ADT + apalutamide	M1 metastases, ECOG 0-1
Hungary [29]	**Metastatic castration-resistant prostate cancer (2nd line/post chemo)**	
	Abiraterone	ECOG 0-1
	Enzalutamide	ECOG 0-1
	**Metastatic castration-resistant prostate cancer (1st line)**	

Country	Description of Restrictions	
Italy [30,31]	*Abiraterone (Zytiga)*	Emilia-Romagna: Recommended with restrictions to treatable population (includes drugs with tracking MEAs or CDF funding).
	Enzalutamide (Xtandi)	Emilia-Romagna: Recommended with restrictions to treatable population (includes drugs with tracking MEAs or CDF funding).
	**Metastatic castration-resistant prostate cancer (2nd line/post chemo)**	
	Abiraterone (Zytiga)	Emilia-Romagna: ECOG 0-1
	Cabazitaxel (Jevtana)	Emilia-Romagna: Not recommended
	Enzalutamide (Xtandi)	Emilia-Romagna: ECOG 0-1
	**Metastatic castration-resistant prostate cancer with symptomatic bone metastases**	
	Radium Ra 223 dichloride	Emilia-Romagna: Recommended with restrictions to treatable population (includes drugs with tracking MEAs or CDF funding).
Poland [32]	**Metastatic castration-resistant prostate cancer (1st line)**	
	Abiraterone	ECOG 0-1, age >18 yr
	Enzalutamide	ECOG 0-1, age >18 yr
	**Metastatic castration-resistant prostate cancer (2nd line/post-chemo)**	
	Abiraterone	ECOG 0-1, age >18 yr
	Enzalutamide	ECOG 0-1, age >18 yr
	**Non-metastatic castration-resistant prostate cancer**	
	ADT + apalutamide	No distant metastases (M0; N1 allowed), PSA doubling time ≤10 months, ECOG 0-1, age >18 yr
	ADT + darolutamide	No distant metastases (M0; N1 allowed), PSA doubling time ≤10 months, ECOG 0-1, age >18 yr
	ADT + enzalutamide	No distant metastases (M0; N1 allowed), PSA doubling time ≤10 months, ECOG 0-1, age >18 yr
	**Metastatic castration-resistant prostate cancer with symptomatic bone metastases**	
	Radium Ra 223 dichloride	Testosterone concentration <50 ng/dL or surgical castration; progression after min. 2 lines of treatment of metastatic castration-resistant disease; OR counter-indications for previous systemic treatment; minimum 6 bone metastases confirmed by scintigraphy; bone pain demanding constant usage of analgesics; ECOG 0-2; >age 18 yr; adequate blood parameters.
Portugal [33]	**Metastatic castration-resistant prostate cancer (1st line)**	
	Enzalutamide	ECOG 0 and 1 only.
	**Non-metastatic castration-resistant prostate cancer**	
	ADT + apalutamide	PS ECOG 0-1; PSA levels duplication <10 months during ADT.
	ADT + darolutamide	PS ECOG 0-1; PSA levels duplication <10 months during ADT.
Slovakia [34]	**Metastatic hormone-sensitive prostate cancer**	
	ADT + abiraterone	In combination with prednisone or prednisolone for treatment of newly diagnosed high risk metastatic, hormone sensitive prostate cancer in adult men in combination with ADT. High risk patient has to meet at least 2 of 3 criteria of high risk: Gleason score ≥8; number of bone lesions ≥3; presence of visceral mets. Treatment is

Country	Description of Restrictions	
		reimbursed until disease progression and has to be preapproved by HIC.
	ADT + apalutamide	For treatment of metastatic mHCPC (mHSPC, metastatic hormone sensitive prostate cancer) in combination with ADT in adult men. Treatment is reimbursed until progression. Insurance company preapproval is required.
	**Metastatic castration-resistant prostate cancer (1st line)**	
	Abiraterone	In combination with prednisone or prednisolone for treatment of metastatic castration-resistant prostate cancer in adult men who are asymptomatic or with mild symptoms after progression on ADT and chemotherapy is not yet clinically indicated. Patients have to be in performance status ECOG 0-1, with no visceral mets, cannot suffer from cancer pain and level of PSA ≤ 114 ng/m, Hgb ≥13 g/dL, PSADT ≥ 55 days. Treatment is reimbursed until clinical progression.
	Enzalutamide	For treatment of metastatic castration-resistant prostate cancer in adult men who are asymptomatic or with mild symptoms after progression on ADT and chemotherapy is not yet clinically indicated. Patients have to be in performance status ECOG 0-1, with no visceral mets, cannot suffer from cancer pain and level of PSA ≤ 114 ng/mL, Hgb ≥ 13 g/dL, PSADT ≥ 55 days. Treatment is reimbursed until clinical progression. Treatment has to be preapproved by HIC.
	**Non-metastatic castration-resistant prostate cancer**	
	ADT + apalutamide	For treatment of nonmetastatic CRPC in combination with ADT in adult men at high risk of developing metastatic disease (PSADT ≤10 months). Treatment is reimbursed until radiologically confirmed progression or un-acceptable toxicity. Treatment has to be preapproved by insurance company.
Slovenia [35]	**Metastatic castration-resistant prostate cancer with symptomatic bone metastases**	
	Radium Ra 223 dichloride	Treatment can be initiated only by oncologist in Oncology Institute of Ljubljana.
Spain [36,37]	**Metastatic hormone-sensitive prostate cancer**	
	ADT + abiraterone	Docetaxel- ineligible
	ADT + apalutamide	Valencia: docetaxel contraindicated
	**Metastatic castration-resistant prostate cancer (1st line)**	
	Abiraterone	No AEMPS IPT; Valencia: ECOG 0-1; Recommended with restrictions to treatable population (includes drugs with tracking MEAs or CDF funding)
	Enzalutamide	Valencia: ECOG 0-1
	**Metastatic castration-resistant prostate cancer (2nd line/post chemo)**	
	Abiraterone	No AEMPS IPT; Valencia: ECOG 0-1; Recommended with restrictions to treatable population (includes drugs with tracking MEAs or CDF funding)
	Cabazitaxel	No AEMPS IPT; Valencia: ECOG 0-1; Recommended with restrictions to treatable population (includes drugs with tracking MEAs or CDF funding)

Country	Description of Restrictions	
	Enzalutamide	Valencia: ECOG 0-1
	**Non-metastatic castration-resistant prostate cancer**	
	ADT + apalutamide	High risk, ECOG 0-1, PSA levels, Valencia: as AEMPS
Sweden [38,39]	**Metastatic hormone-sensitive prostate cancer**	
	ADT + apalutamide	Subsidized only for the treatment of adult men with (1) non-metastatic castration-resistant prostate cancer (nmCRPC) who are at high risk of developing metastatic disease, (2) metastatic hormone-sensitive prostate cancer (mHSPC) in combination with androgen deprivation therapy (ADT).
	**Metastatic castration-resistant prostate cancer (1st line)**	
	Enzalutamide	Xtandis 2nd indication. Restricted reimbursement for both indications Nov 2017.
	**Metastatic castration-resistant prostate cancer (2nd line/post chemo)**	
	Cabazitaxel	Only use cabazitaxel to treat metastatic prostate cancer to the group that initially responded but progressed within 3 months of the last dose of docetaxel. This given that the agreement on lower price offered by the company is used.
	**Non-metastatic castration-resistant prostate cancer**	
	ADT + apalutamide	Subsidized only for the treatment of adult men with 1) non-metastatic castration-resistant prostate cancer (nmCRPC) who are at high risk of developing metastatic disease, 2) metastatic hormone-sensitive prostate cancer (mHSPC) in combination with androgen deprivation therapy (ADT).
	**Metastatic castration-resistant prostate cancer with symptomatic bone metastases**	
	Radium Ra 223 dichloride	Combination of radium-223 with abiraterone and prednisolone significantly increases the risk of fractures (478). This has led to the Medical Products Agency limiting the indication for radium-223 and strengthening the requirement for follow-up of skeletal status and treatment to reduce fracture risk (see Indication and Fracture Prophylaxis in chapter 13.3.6.1 in the attached National care program). For men at high risk of fractures, the possible benefit of radium-223 should be weighed against a possible, treatment-related risk increase for fractures.
Switzerland [40,41]	**Metastatic hormone-sensitive prostate cancer**	
		For treatment in combination with LHRH agonists and prednisone or prednisolone in asymptomatic or mildly symptomatic patients with metastatic castration-resistant prostate cancer (mCRPC) without visceral metastases and without liver metastases, after failure of androgen deprivation therapy, when chemotherapy is not clinically indicated.<br><br>For treatment in combination with LHRH agonists and prednisone or prednisolone in patients with advanced metastatic prostate carcinoma in progression after treatment with docetaxel after cost approval by the health insurer and prior consultation with the medical officer. Treatment in combination with prednisone or prednisolone (5 mg/day) and androgen deprivation therapy (ADT) in patients newly diagnosed with high-risk metastatic hormone-sensitive prostate cancer (mHSPC) within the last 3 months. Abiraterone should be

Country	Description of Restrictions	
		started within 3 months of the start of androgen deprivation in non-orchiectomized patients.<br><br>High risk is defined as the presence of at least 2 of the following 3 risk factors: (1) Gleason score of ≥8; (2) presence of ≥3 lesions on bone scan; (3) presence of measurable visceral metastases (not including lymph node involvement). In the event of a discontinuation of therapy within 10 days of the start of treatment due to side effects, the costs of the entire pack will be reimbursed to the health insurer by Janssen-Cilag AG. The treatment discontinuation must be reported to the health insurer immediately by the attending physician.
	ADT + enzalutamide (XTANDI)	After approval of costs by the health insurer with prior consultation of the medical officer. XTANDI is reimbursed in combination with LHRH agonists for the treatment of men with metastatic castration-resistant prostate cancer (CRPC) with an asymptomatic or mildly symptomatic course after failure of androgen deprivation therapy, and for whom chemotherapy is not yet clinically indicated. XTANDI is reimbursed in combination with LHRH agonists for the treatment of men with metastatic castration-resistant prostate cancer in progression under or after docetaxel therapy. Limitation until 31.03.2023: XTANDI is reimbursed in combination with LHRH agonists for the treatment of men with metastatic hormone-sensitive prostate cancer (mHSPC). In the event of a discontinuation of therapy within 10 days of the start of treatment due to side effects, the costs of the entire pack will be reimbursed to the health insurer by Astellas Pharma AG. The treatment discontinuation must be reported to the health insurer immediately by the attending physician.
	**Metastatic castration-resistant prostate cancer (1st line)**	
	Abiraterone	For treatment in combination with LHRH agonists and prednisone or prednisolone in asymptomatic or mildly symptomatic patients with metastatic castration-resistant prostate cancer (mCRPC) without visceral metastases and without liver metastases, after failure of androgen deprivation therapy, when chemotherapy is not clinically indicated.<br><br>For treatment in combination with LHRH agonists and prednisone or prednisolone in patients with advanced metastatic prostate carcinoma in progression after treatment with docetaxel after cost approval by the health insurer and prior consultation with the medical officer. Treatment in combination with prednisone or prednisolone (5 mg/day) and androgen deprivation therapy (ADT) in patients newly diagnosed with high-risk metastatic hormone-sensitive prostate cancer (mHSPC) within the last 3 months. Abiraterone should be started within 3 months of the start of androgen deprivation in non-orchiectomized patients.<br><br>High risk is defined as the presence of ≥2 of the following 3 risk factors: (1) Gleason score of ≥8; (2) presence of at least 3 lesions on bone scan; (3) presence of measurable visceral metastases (not including lymph node involvement). In the event of a discontinuation of therapy within 10 days of the start of treatment

Country	Description of Restrictions	
		due to side effects, the costs of the entire pack will be reimbursed to the health insurer by Janssen-Cilag AG. The treatment discontinuation must be reported to the health insurer immediately by the attending physician.
	Enzalutamide	After approval of costs by the health insurer with prior consultation of the medical officer. XTANDI is reimbursed in combination with LHRH agonists for the treatment of men with metastatic castration-resistant prostate cancer (CRPC) with an asymptomatic or mildly symptomatic course after failure of androgen deprivation therapy, and for whom chemotherapy is not yet clinically indicated. XTANDI is reimbursed in combination with LHRH agonists for the treatment of men with metastatic castration-resistant prostate cancer in progression under or after docetaxel therapy.<br><br>Limitation until 31.03.2023: XTANDI is reimbursed in combination with LHRH agonists for the treatment of men with metastatic hormone-sensitive prostate cancer (mHSPC).<br><br>In the event of a discontinuation of therapy within 10 days of the start of treatment due to side effects, the costs of the entire pack will be reimbursed to the health insurer by Astellas Pharma AG. The treatment discontinuation must be reported to the health insurer immediately by the attending physician.
	**Metastatic castration-resistant prostate cancer (2nd line/post chemo)**	
	Abiraterone	For treatment in combination with LHRH agonists and prednisone or prednisolone in asymptomatic or mildly symptomatic patients with metastatic castration-resistant prostate cancer (mCRPC) without visceral metastases and without liver metastases, after failure of androgen deprivation therapy, when chemotherapy is not clinically indicated.<br><br>For treatment in combination with LHRH agonists and prednisone or prednisolone in patients with advanced metastatic prostate carcinoma in progression after treatment with docetaxel after cost approval by the health insurer and prior consultation with the medical officer. Treatment in combination with prednisone or prednisolone (5 mg/day) and androgen deprivation therapy (ADT) in patients newly diagnosed with high-risk metastatic hormone-sensitive prostate cancer (mHSPC) within the last 3 months. Abiraterone should be started within 3 months of the start of androgen deprivation in non-orchiectomized patients.<br><br>High risk is defined as the presence of ≥2 of the following 3 risk factors: (1) Gleason score of ≥8; (2) presence of at least 3 lesions on bone scan; (3) presence of measurable visceral metastases (not including lymph node involvement). In the event of a discontinuation of therapy within 10 days of the start of treatment due to side effects, the costs of the entire pack will be reimbursed to the health insurer by Janssen-Cilag AG. The treatment discontinuation must be reported to the health insurer immediately by the attending physician.

Country	Description of Restrictions	
	Cabazitaxel	In combination with prednisone or prednisolone for the treatment of metastatic castration-resistant prostate cancer (mCRPC) in patients who have previously received chemotherapy with docetaxel, if these patients have not responded to docetaxel or if the carcinoma has shown progression within 6 months after the end of therapy with docetaxel.<br><br>The reimbursement of the treatment requires the approval of the health insurer after prior consultation with the medical officer.<br>A maximum of 10 cycles are to be reimbursed. If the therapy is discontinued before the start of the 2nd cycle of Jevtana, Sanofi-Aventis (Suisse) SA will reimburse the costs of the dose of Jevtana administered during the 1st cycle to the payer at the request of the insurer.
	Enzalutamide	After approval of costs by the health insurer with prior consultation of the medical officer. XTANDI is reimbursed in combination with LHRH agonists for the treatment of men with metastatic castration-resistant prostate cancer (CRPC) with an asymptomatic or mildly symptomatic course after failure of androgen deprivation therapy, and for whom chemotherapy is not yet clinically indicated. XTANDI is reimbursed in combination with LHRH agonists for the treatment of men with metastatic castration-resistant prostate cancer in progression under or after docetaxel therapy.<br><br>Limitation until 31.03.2023: XTANDI is reimbursed in combination with LHRH agonists for the treatment of men with metastatic hormone-sensitive prostate cancer (mHSPC). In the event of a discontinuation of therapy within 10 days of the start of treatment due to side effects, the costs of the entire pack will be reimbursed to the health insurer by Astellas Pharma AG. The treatment discontinuation must be reported to the health insurer immediately by the attending physician.
	**Metastatic castration-resistant prostate cancer with symptomatic bone metastases**	
	Radium Ra 223 dichloride	For the treatment of patients with castration-resistant prostate cancer (CRPC) and symptomatic bone metastases without known visceral metastases when chemotherapy is not indicated or in case of progression after docetaxel and not concomitantly with 2nd-generation androgen deprivation therapy (ADT) (for example, abiraterone acetate and enzalutamide) and not with cabazitaxel.<br><br>A maximum of 6 cycles of therapy will be reimbursed. In non-orchiectomized patients, antiandrogenic treatment to suppress testosterone levels to castration levels should be continued.
United Kingdom [42]	**Metastatic castration-resistant prostate cancer (2nd line/post chemo)**	
	Abiraterone	>1 docetaxel-containing regimen (with MEA)
	Cabazitaxel	ECOG 0-1, quantity limit, (with PAS and MEA)
	**Metastatic castration-resistant prostate cancer with symptomatic bone metastases**	

Country	Description of Restrictions	
	Radium Ra 223 dichloride	Postdocetaxel or docetaxel contraindicated (with PAS)

Abbreviations: ADT: androgen deprivation therapy; AEMPS: Spanish Agency of Medicines and Medical Devices; BPI: Brief Pain Inventory; BRCA: BReast CAncer gene; CDF: Cancer Drug Fund; CRPC: castration-resistant prostate cancer; CT: computed tomography; dl: deciliter; ECOG: Eastern Cooperative Oncology Group; HIC: Hofmann International Consulting; IPT: Insurance Premium Tax; kBq kilobecquerel; kg: kilogram; l: liter; LHRH: luteinizing hormone-releasing hormone; m: meter; mCRPC: metastatic castration-resistant prostate cancer; MEA: managed entry agreements; mg: milligram; mHSPC: metastatic hormone sensitive prostate cancer; ml: milliliter; ng: nanogram; nmCRPC: non-metastatic castration-resistant prostate cancer; nmol: nanomole; NMR: nuclear magnetic resonance; PAS: Patient Access Scheme; PS: Performance status; PSA: prostate specific antigen; PSA-DT: prostate specific antigen doubling time; RECIST: Response Evaluation Criteria in Solid Tumors; SF: Short Form.
